# DeepMEns: an ensemble model for predicting sgRNA on-target activity based on multiple features

**DOI:** 10.1093/bfgp/elae043

**Published:** 2024-11-11

**Authors:** Shumei Ding, Jia Zheng, Cangzhi Jia

**Affiliations:** School of Science, Dalian Maritime University, Dalian 116026, China; School of Science, Dalian Maritime University, Dalian 116026, China; School of Science, Dalian Maritime University, Dalian 116026, China

**Keywords:** CRISPR/Cas9, CNN, attention mechanism, integration algorithm, sgRNA, position encoding

## Abstract

The CRISPR/Cas9 system developed from *Streptococcus pyogenes* (SpCas9) has high potential in gene editing. However, its successful application is hindered by the considerable variability in target efficiencies across different single guide RNAs (sgRNAs). Although several deep learning models have been created to predict sgRNA on-target activity, the intrinsic mechanisms of these models are difficult to explain, and there is still scope for improvement in prediction performance. To overcome these issues, we propose an ensemble interpretable model termed DeepMEns based on deep learning to predict sgRNA on-target activity. By using five different training and validation datasets, we constructed five sub-regressors, each comprising three parts. The first part uses one-hot encoding, wherein 0–1 representation of the secondary structure is used as the input to the convolutional neural network (CNN) with Transformer encoder. The second part uses the DNA shape feature matrix as the input to the CNN with Transformer encoder. The third part uses positional encoding feature matrices as the proposed input into a long short-term memory network with an attention mechanism. These three parts are concatenated through the flattened layer, and the final prediction result is the average of the five sub-regressors. Extensive benchmarking experiments indicated that DeepMEns achieved the highest Spearman correlation coefficient for 6 of 10 independent test datasets as compared to previous predictors, this finding confirmed that DeepMEns can accomplish state-of-the-art performance. Moreover, the ablation analysis also indicated that the ensemble strategy may improve the performance of the prediction model.

## Introduction

The CRISPR/Cas9 system developed from *Streptococcus pyogenes* (SpCas9) has high potential in gene editing. The combination of artificially designed CRISPR RNA (crRNA) and trans-activating crRNA (tracrRNA) can form single guide RNA (sgRNA) with a guiding effect, which directs the Cas9 protein to perform precise cleavage of the target DNA sequence, thereby preparing for the specific gene editing task [1]. Previous studies have indicated that the targeting process of CRISPR/Cas9 is not only influenced by the sequence characteristics of sgRNA but also affected by some local 3D structural factors and the functional status of target genes [2, 3]. Therefore, the accurate prediction of sgRNA activity will help improve the experimental safety based on CRISPR systems.

With the advancement of computational technology, various sgRNA design rules and tools have been proposed to recognize and predict the efficacy of sgRNA targeting. The most available deep learning-based approaches for sgRNA on-target activity prediction are based on convolutional neural network (CNN), recurrent neural network (RNN), or their combination. The techniques for converting sgRNA sequences into feature vectors primarily involve one-hot encoding, shape features (rolling (Roll), propeller twist (ProT), minor groove width (MGW), helix twist (HeIT), and electrostatic potential (EP)], hand-crafted biological features (GC content features, thermodynamics features, Zcurve, (A + T)/(C + G) ration, GC/AT skew, and so on), and epigenetic characteristics. DeepCRISPR [4] is believed to be the earliest model to employ deep learning methods for predicting CRISPR sgRNA on-target activity for Cas9, this model utilizes one-hot encoding and epigenetic features such as DTDF, RRBS, H3K4me3, and Dnase for characterizing sgRNA sequences. Subsequent to DeepHF [5], DeepCas9 [6], DeepSgrnaBacteria [7], AttnToCrispr_CNN [8], and DeepSpCas9 [9] were introduced in 2019, with most of these models relying on one-hot encoding and the CNN algorithm. Nonetheless, these models are based on distinct datasets. DeepHF [5] was designed to measure sgRNA activity for two highly specific SpCas9 variants (eSpCas9(1.1) and SpCas9-HF1) and the wild-type SpCas9 (WT-SpCas9) through a genome-scale screen, and three large-scale benchmark datasets (WT-SpCas9, eSpCas9(1.1), and SpCas9-HF1) were established. DeepSpCas9 [9] was designed and evaluated on the HT_Cas9_Train and HT_Cas9_Test datasets containing 12 832 target sequences in a human cell. DeepSgrnaBacteria [7] was designed to predict sgRNA activity in *Escherichia coli* by using a CNN. Subsequently, many deep learning-based methods have been proposed, including DeepxCas9 [10], CRISPRon [11], CRISPR-ONT [12], sgRNACNN [13], AttCRISPR [14], CNN-SVR [15], CNN-XG [16], TransCrispr [17], and DeepCRISTL [18], all of them have achieved excellent results in the field of sgRNA activity prediction. The recent reviews [19–21] have provided detailed descriptions of each of these models. CRISeq [22] was proposed in 2023 and developed based on a fusion framework of CNN, RNN, and Light Gradient Boosting Machine (LightGBM) [23]. It adopts the datasets in DeepHF [5] to train the model and was tested on 10 independent datasets. More recently, Zhang *et al.* constructed CrnnCrispr [24], a hybrid network framework that combines CNN and Bidirectional Gated Recurrent Unit and utilizes transfer learning for predicting sgRNA on-target activity.

The aforementioned studies suggest that appropriately enhancing the model’s complexity will improve its ability to identify sgRNA activity to varying extents. Here, the ensemble strategy was considered to further boost the model’s performance in identifying sgRNA activity. An ensemble learning approach was employed to develop DeepMEns using five different training and validation datasets from DeepHF [5]. Subsequently, five sub-regressors were constructed, each comprising three components. Specifically, the first and second components involve one-hot encoding combined with a 0–1 representation of the secondary structure feature obtained by RNAFold in ViennaRNA [25] and a DNA shape feature matrix, respectively, fed into the CNN with Transformer encoder [26]. The third component utilizes the positional encoding feature matrices we proposed as input into a long short-term memory network with an attention mechanism. These three parts are concatenated through the flatten layer and inputted into the dense layer. The final prediction result is the average of the five sub-regressors. Extensive benchmarking experiments indicated that the ensemble neural networks showed the highest Spearman correlation coefficient of 0.880 and 0.875 for WT-SpCas9 and eSpCas9(1.1), respectively, which was 0.008 and 0.008, respectively, higher than the second-best predictor AttCRISPR [14]. Furthermore, our model clearly outperformed other models on 6 of 10 datasets. Ablation analysis showed that the ensemble strategy may improve the performance of the prediction model.

## Material and methods

### Benchmark datasets

Initially, we utilized the benchmark datasets constructed by Wang *et al.* [5], which have been widely referenced in numerous studies [27]. Wang *et al.* performed a genome-scale screening to evaluate the activity of sgRNAs for a WT-SpCas9 as well as two highly specific SpCas9 variants, eSpCas9(1.1) and SpCas9-HF1, in human cells. The datasets comprise 55,604, 58,617, and 56,888 sgRNAs, with their activities quantified by insertion/deletion (indel) events associated with WT-SpCas9, eSpCas9(1.1), and SpCas9-HF1, respectively. We subsequently partitioned this data into training and testing subsets in an 85:15 ratio. Drawing on insights from previous research, the training datasets were employed to optimize and develop the model, while the testing datasets served to assess the predictive performance of the trained model. Moreover, we also collected 10 additional independent datasets derived from various cell lines and species to further assess the generalizability of our prediction model [28–35]. The criteria employed to measure the effectiveness of sgRNA splicing varied across these datasets, some relied on actual read counts or fold changes, while others focused on the ratio of successful edits. To evaluate the model’s performance, we applied both Spearman and Pearson correlation coefficients. This approach allows us to analyze the relationship between two variables without the effects of different measurement units, thus negating the need for standardization of the diverse criteria. For ease of reference, Table 1 presents a statistical analysis and summary of the aforementioned datasets. All training and test datasets are accessible at https://github.com/Dingshumei/DeepMEns.git.

**Table 1:** Statistics of 14 datasets.

Datasets	^a^Name	Biological material	Size	sgRNA length	Efficiency format	Experimental conditon
Training dataset	^5^Daqi Wang_Human_2019 (WT-SpCas9)	HEK293T/Hela cell	55,604	21	Percentage	Lentivirus transfection
	^5^Daqi Wang_Human_2019 (eSpCas9(1.1))	HEK293T/Hela cell	58,617	21	Percentage	Lentivirus transfection
	^5^Daqi Wang_Human_2019 (SpCas9-HF1)	HEK293T/Hela cell	56,888	21	Percentage	Lentivirus transfection
	^9^Hui Kwon Kim_Human_2019 (HT_Cas9)	HEK293T	13,374	21	Percentage	Endogenous
Independent dataset	^28^Tim Wang_human_2014	Hl60 cell	2076	21	Percentage	Endogenous
	^29^Xingjie Ren_Fruitfly_2014	Drosophila syncitial embryos	39	21	Normalized Percentile	Endogenous
	^30^Moreno A Moreno-Mateos_Zebrafish_2015	Zebrafish embryos	1020	21	Normalized Percentile	Endogenous
	^31^Traver Hart _Human_2016	HCT116 cell	4172	20	Log Fold Change	Endogenous
	^31^Traver Hart_Human_2016	Hela cell	3566	20	Log Fold Change	Endogenous
	^32^John G Doench_Human_2014	MOLM13/NB4 cell	881	21	Percentage	Endogenous
	^32^John G Doench _Mouse_2014	EL4 cell	945	21	Percentage	Endogenous
	^33^John G Doench _Human_2016	A375/HEK293T/MOLM13 cell	2332	20	Log Fold Change	Endogenous
	^34^Raj Chari_Human_2015	HEK293T cell	1150	21	Log Fold Change	Lentivirus transfection
	^35^Gaurav K Varshney_Zebrafish_2015	Zebrafish embryos	88	21	Normalized Percentile	Endogenous

^a^Note: Author_Species _Year

### Feature encoding

Characterization of sgRNA sequence information was conducted based on three aspects, the primary sequence itself, spatial structure, and positional information. One-hot encoding was employed to represent the sgRNA sequence due to its simplicity of operation and ease of comprehension. The three-dimensional structure of DNA is instrumental in determining the binding preferences of DNA-binding proteins [36]. Consequently, five shape features of DNA were introduced for the first time in the field of gene editing. Secondary structure serves as a significant intermediate level of nucleotide description, encompassing the dominant portion of folding energy and playing a pivotal role in providing sequence information [20]. To account for variations in nucleotide arrangement orders, a novel position information encoding was utilized. Additionally, six hand-crafted biological features [37–40], which may enhance the predictive power of the model, were selected (Table S1).

#### One-hot encoding

The one-hot encoding is adopted to seize the content and order information of nucleotides in sgRNA sequences [41]. Each sgRNA sequence contains four nucleotides A, G, C, and T. As usual, ‘A’ is encoded as (1, 0, 0, 0), ‘C’ is encoded as (0, 1, 0, 0), ‘G’ is encoded as (0, 0, 1, 0), and ‘T’ is encoded as (0, 0, 0, 1). Thus, according to one-hot encoding, the sgRNA sequence was transformed to a matrix ${M}_1$ of $21\times 4$, where 21 is the length of each sgRNA sequence.

#### DNA shape feature

The five shape features [42] include HelT, MGW, ProT, Roll, and minor groove EP are adopted to depict spatial pose. In addition, these features are easily accessible from DNAshapeR, which is a R package for ultra-fast, high-throughput prediction of DNA shape features. As a result, the DNA shape features for the sgRNA sequence input are represented as a feature matrix ${M}_2$ of 21 × 5, where 21 is the length of each sgRNA sequence.

#### Secondary structure feature

The spatial structure of RNA is very complex and difficult to obtain through experimental methods. Then, we obtain the RNA secondary structure predicted by RNAFold in the ViennaRNA [25]. RNA sequences are written from left to right, while base pairing is indicated by parentheses. The matching left and right parentheses represent a pair of base pairs, while dots represent unpaired bases. For instance, the secondary structure of ‘AAAAAACAGATGCCACCTGTG’ is predicted as ‘ …..(((( … ….)))).’. We represent ‘(’ and ‘)’ as 1 and ‘.’ as 0. For example, ‘ …..(((( … ….)))).’ is defined as ‘000001111000000011110’. By this way, the secondary structure feature for the sgRNA sequence input is represented as a feature vector ${M}_3$ with the dimension of 21 × 1.

#### Position encoding

**Figure 1:**
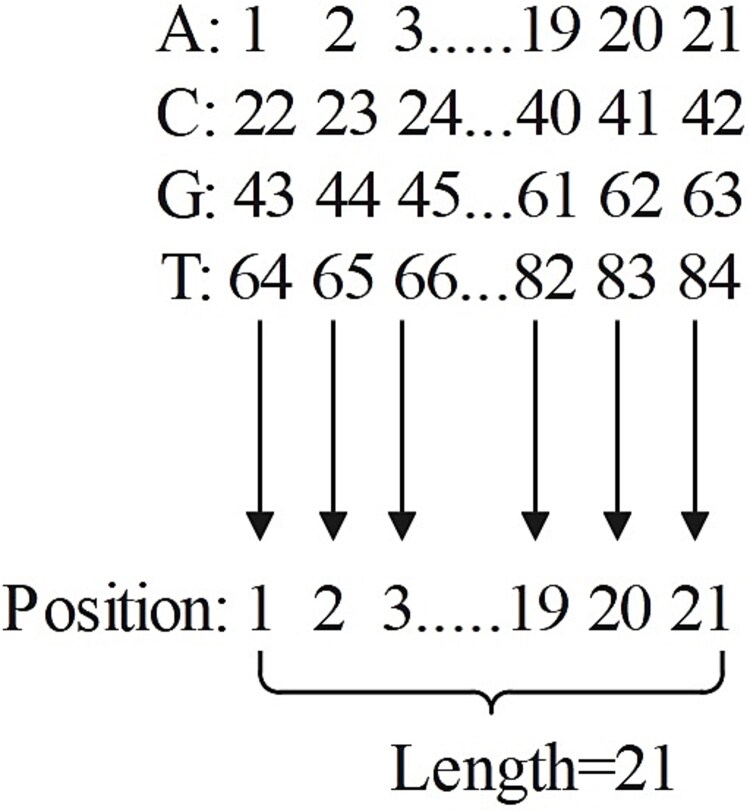
Encoding of each nucleotide at different positions.

We propose a new position encoding by assigning each nucleotide a different value based on its position as shown in Fig. 1. For instance, ‘AAAAAACAGATGCCACCTGTG’ is encoded as ‘1, 2, 3, 4, 5, 6, 28, 8, 51, 10, 74, 54, 34, 35, 15, 37, 38, 81, 61, 83, 63’. Thus, the position encoding for the sgRNA sequence input are represented as a vector ${M}_4$ of 21 × 1.

### Architecture of DeepMEns

**Figure 2:**
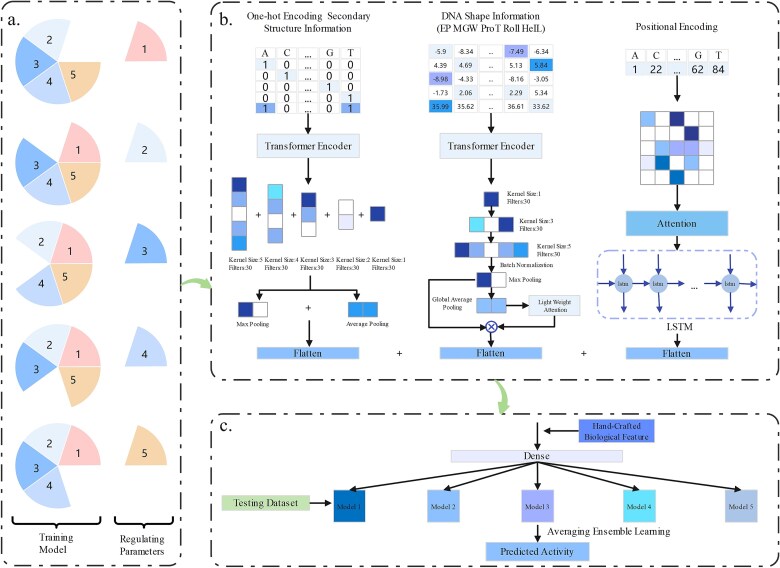
The overall framework of DeepMEns. (a) Partitioning of the training datasets. The training dataset is further partitioned into five parts where one part is used to regulate the hyper-parameters, and the remaining four parts are used for training. (b) There are three neural network frameworks. The first framework processes one-hot encoding combined with 0–1 representation of the secondary structure feature. The second framework processes the DNA shape feature matrix. The third framework processes the positional encoding feature matrices. (c) The independent test datasets are inputted into the five models with different weights and biases to compare the performance by using the average ensemble learning approach.

We adopted the ensemble learning strategy to develop DeepMEns based on five different training and validation datasets. As shown in Fig. 2(b), DeepMEns mainly comprise three parts. In the first part, the combination of one-hot encoding and 0–1 representation of the secondary structure feature is fed into the CNN with Transformer encoder [26] to extract high-dimensional feature representation. The convolution layer contains five different sizes of filters (1-nt, 2-nt, 3-nt, 4-nt, and 5-nt filters), and the number of filters is set as 30. The rectified linear unit (Relu) activation is used in the convolution layer. Max pooling and average pooling are adopted in the pooling layer. Technically, the module operations are summarized as follows:


(1) \begin{equation*} {a}_0= TransformerEncoder\left({M}_1{M}_3\right), \end{equation*}

(2) \begin{equation*} {a}_i= Relu\left({Conv}_i\left({a}_0\right)\right),i=1,2,3,4,5, \end{equation*}

(3) \begin{align*} a=&\ Flatten\left( MaxPooling\left({a}_1+{a}_2+{a}_3+{a}_4+{a}_5\right)\right. \nonumber \\ &\left. + AveragePooling\left({a}_1+{a}_2+{a}_3+{a}_4+{a}_5\right)\right). \end{align*}

The DNA shape feature matrix is also used as the input for the CNN with Transformer encoder [26]. The convolution layer includes three different sizes of filters (301-nt, 303-nt, and 30 5-nt filters) through the grid search and a LightWeight attention mechanism [43]. Max pooling and global average pooling are used. Relu activation is adopted in the convolution layer. The calculation is as follows:


(4) \begin{equation*} {b}_0= TransformerEncoder\left({M}_2\right), \end{equation*}

(5) \begin{equation*} {b}_j= Relu\left({Conv}_j\left({b}_0\right)\right),i=1,2,3, \end{equation*}

(6) \begin{equation*} {b}_4= MaxPooling\left( BatchNormalization\left({b}_3\left({b}_2\left({b}_1\right)\right)\right)\right), \end{equation*}

(7) \begin{equation*} {b}_5= LightWeightAttention\left( GlobalAveragePooling\left({b}_4\right)\right), \end{equation*}

(8) \begin{equation*} b= Flatten\left({b}_4\otimes{b}_5\right), \end{equation*}

The positional encoding feature matrices are used as the input for the long short-term memory (LSTM) network with an attention mechanism [26] to extract long-distance dependencies and implicit relationships among the sgRNA sequence. Technically, the operation described above is as follows:


(9) \begin{equation*} c= Flatten\left( LSTM\left( Attention\left( Embedding\left({M}_4\right)\right)\right)\right), \end{equation*}

Next, the feature represention vectors a, b, and c from three parts are concatenated with hand-crafted biological feature vector to provide input to a fully connected layer.

DeepMEns encompasses various hyperparameters that require optimization, including the number of filters in the convolutional layer, the size of the convolutional kernel, the units in BiLSTM, and the choice of optimizer. To identify the optimal hyperparameters, we employ grid search within a specified space and utilize the Spearman correlation coefficient as our evaluation metric. Table S2 presents the search space and final values for each category of hyperparameters. Derived from five distinct training and validation datasets, five base regression models were formulated, and their collective average determined the final decision. The methods proposed were executed using Python 3.7 and Keras (2.10.0) alongside TensorFlow (2.4.1). Within the Keras class library lie various optimizers capable of dynamically adjusting the learning rate size. Throughout the training phase, early stopping and the adaptive moment estimation (Adam) optimizer [44] were applied to enhance generalizability, hasten convergence, and prevent overfitting. Mean square error (MSE) served as the designated loss function for the regression task.

### Evaluation metrics

The performance is evaluated using three commonly evaluation metrics, including the Spearman correlation coefficient ($\mathrm{\rho}$), Pearson correlation coefficient ($\mathrm{\gamma}$), and MSE:


(10) \begin{equation*} \rho =1-\frac{6\sum_{i=1}^n{\left(R\left({Y}_i\right)-R\left(\hat{Y_i}\right)\right)}^2}{n\left({n}^2-1\right)}, \end{equation*}

(11) \begin{equation*} \gamma =\frac{\sum_{i=1}^n\left({Y}_i-\overline{Y}\right)\left(\hat{Y_i}-\hat{\overline{Y_i}}\right)}{\sqrt{\sum_{i=1}^n{\left({Y}_i-\overline{Y}\right)}^2}\sqrt{\sum_{i=1}^n{\left(\hat{Y_i}-\hat{\overline{Y_i}}\right)}^2}}, \end{equation*}

(12) \begin{equation*} MSE=\frac{\sum_{i=1}^n{\left({Y}_i-\hat{Y_i}\right)}^2}{n}, \end{equation*}

where *n* is the number of samples, ${Y}_i$ is the raw score, $\hat{Y_i}$ is the predictive score, $R\left({Y}_i\right)$ and $R\left(\hat{Y_i}\right)$ are rank transformation of ${Y}_i$ and $\hat{Y_i}$, and $\overline{Y}$ and $\hat{\overline{{\mathrm{Y}}_{\mathrm{i}}}}$ are the sample mean, respectively.

## Results and discussion

### The averaging ensemble scheme improves prediction performance

**Figure 3:**
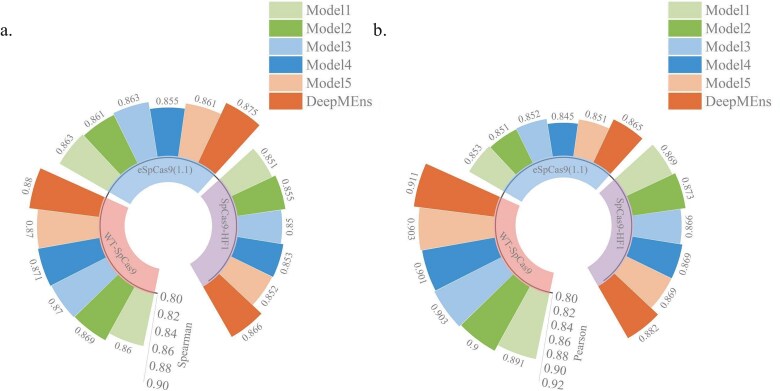
Results of comparison of DeepMEns with five base regression models on the independent test datasets of WT-SpCas9, eSpCas9(1.1), and SpCas9-HF1. (a) The comparison of Spearman correlation coefficient. (b) The comparison of Pearson correlation coefficient.

**Figure 4:**
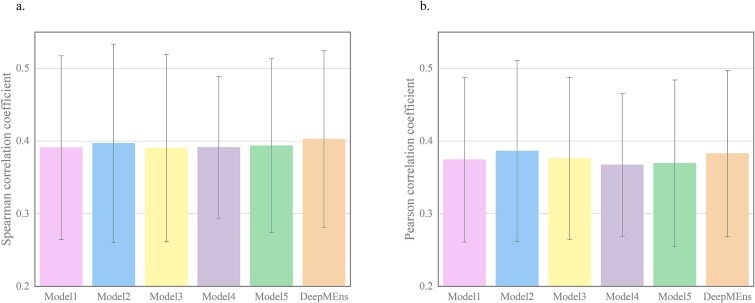
Comparison of the average performance of DeepMEns with five base regression models on 10 independent test datasets. (a) The comparison of Spearman correlation coefficient. (b) The comparison of Pearson correlation coefficient.

To assess the effectiveness of the averaging ensemble approach, we computed the Spearman correlation coefficient, Pearson correlation coefficient, and MSE for DeepMEns alongside its base regression models. As illustrated in Table S3 and Fig. 3, the averaging ensemble method significantly enhances predictive performance. Notably, DeepMEns demonstrated superior results on the independent test datasets for WT-SpCas9, eSpCas9(1.1), and SpCas9-HF1, achieving Spearman scores of 0.880, 0.875, and 0.866, Pearson scores of 0.911, 0.865, and 0.882, and MSE values of 0.0081, 0.0087, and 0.0095, respectively. The five baseline regression models exhibited comparable performances, with Spearman scores ranging from 0.851 to 0.871, Pearson scores between 0.845 and 0.903, and MSEs spanning 0.0088 to 0.0106 across all three independent test datasets. Furthermore, we evaluated DeepMEns on 10 independent test datasets to examine its generalization capability, with results detailed in Fig. 4 and Table S4. Across 13 independent test datasets, DeepMEns outperformed the five base models. Among these models, Model 2 and DeepMEns recorded nearly equivalent average Spearman scores of 0.397 and 0.403, respectively. However, DeepMEns exhibited a notably lower standard deviation, as indicated in Table S4. In addition, we performed Fisher least-significant difference test [45] with a confidence interval of 0.05 to assess whether there are statistically significant differences in the performance of the five base models on ten independent test datasets. The P-values for the Spearman and Pearson correlation coefficients are 0.999 and 0.996, respectively, indicating no significant differences among the five base models. This similar performance can largely be attributed to the similarities in their datasets and parameter configurations. Furthermore, it is evident that each base model contributes positively, and the combination of these five models helps to decrease variance while enhancing the robustness and generalization capabilities of DeepMEns.

**Figure 5:**
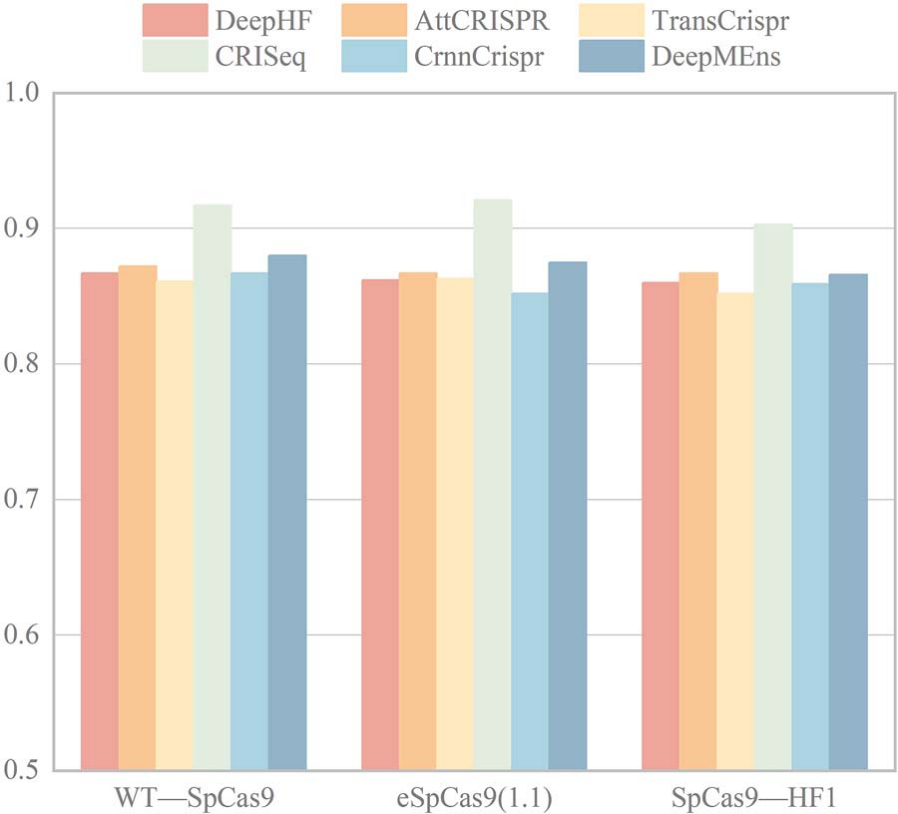
Comparison of performance of various deep learning-based models based on the WT-SpCas9, eSpCas9(1.1), and SpCas9-HF1 test datasets according to Spearman correlation coefficient.

Furthermore, we are evaluating the possibility of substituting deep learning models with conventional machine learning algorithms, including Linear Regression, XGBoost, LightGBM, Random Forest, Support Vector Regression, and AdaBoost. The models will utilize their default parameter configurations. As illustrated in Table 2, there are notable differences in the performance metrics of the six traditional machine learning models. Among them, XGBoost achieves the highest Spearman correlation coefficients of 0.833, 0.810, and 0.800 for the WT-SpCas9, eSpCas9(1.1), and SpCas9-HF1 datasets, respectively. The advantages of DeepMEns may stem from several factors: (1) its multi-layer neural network architecture, which captures intricate multi-level data characteristics, (2) its capability to extract long-range dependencies and implicit relationships from primary features, thereby enhancing the aggregation of valuable information, (3) its superior representational capacity compared to traditional methods, allowing it to model complex nonlinear relationships effectively, and (4) the greater computational resources and time required for iterative processes in DeepMEns compared to classical techniques.

**Table 2:** A comparative analysis of substituting traditional regression techniques for deep learning in the left branch of DeepMEns,

Method	WT-SpCas9	eSpCas9(1.1)	SpCas9-HF1
Linear Regression	0.703	0.661	0.647
XGBoost	0.833	0.810	0.800
LightGBM	0.813	0.779	0.771
Random Forest	0.672	0.587	0.568
SVR	0.816	0.808	0.799
AdaBoost	0.505	0.394	0.383
DeepMEns	0.858	0.852	0.841

### Comparison of DeepMEns with the existing predictors

To further demonstrate the advancement of DeepMEns, we compared it with DeepHF, AttCRISPR, TransCrispr, CRISeq, and CrnnCrispr. The above mentioned model training, validation, and testing are all based on the WT-SpCas9, eSpCas9(1.1), and SpCas9-HF1 datasets. According to the experimental results for the three datasets provided in [5, 14, 17, 22, 24], DeepMEns achieved the second-highest Spearman score for both the WT-SpCas9 and eSpCas9(1.1) datasets, as shown in Fig. 5, falling short of CRISeq by only 0.037 and 0.046, respectively. Additionally, for the SpCas9-HF1 dataset, it secured the third-highest Spearman score, 0.037 lower than CRISeq and 0.001 lower than AttCRISPR. It is important to note that while all data sources are derived from [5], the training and validation datasets are not fully aligned.

**Figure 6:**
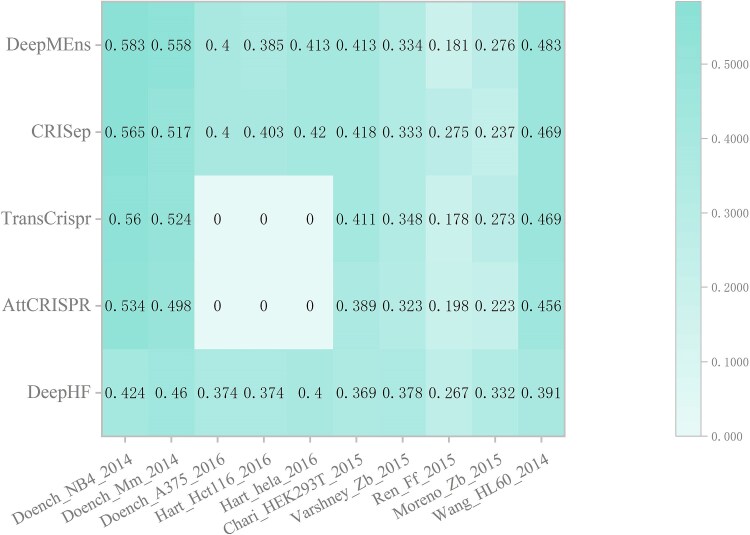
Performance comparison among various deep learning-based models on 10 independent test datasets using Spearman correlation coefficient.

Next, we compared DeepMEns with the state-of-the-art predictors DeepHF, AttCRISPR, TransCrispr, and CRISeq on 10 independent test datasets. Because CrnnCrispr has no source code, we could not verify its performance on 10 independent test datasets. To ensure a fair comparison, the five models were trained on the same dataset WT-SpCas9. AttCRISPR and TransCrisp contain sequences of 21 nucleotides in length, which do not match with the length of sequences in Doench_A375_2016, Hart_Hct116_2016, and Hart_hela_2016. For the purpose of comparing, we set these 6 values to 0 in the analysis. As shown in Fig. 6, DeepMEns achieved the highest Spearman score on 5 independent test datasets, while CRISeq exhibited superior performance on 4 independent test datasets, both models showed the same performance on Doench_A375_2016. For Doench_NB4_2014, Doench_Mm_2014, Varshney_Zb_2015, Moreno_Zb_2015, and Wang_HL60_2014, the Spearman score of DeepMEns exceeded that of CRISeq by 0.018, 0.041, 0.001, 0.039, and 0.014, respectively. For Hart_Hct116_2016, Hart_hela_2016, Chari_HEK293T_2015, and Ren_Ff_2015, Spearman score of CRISeq exceeded that of DeepMEns by 0.018, 0.007, 0.005, and 0.094, respectively. Specifically, there were only 39 sgRNA sequences in Ren_Ff_2015 that could also increase the prediction disparity.

**Figure 7:**
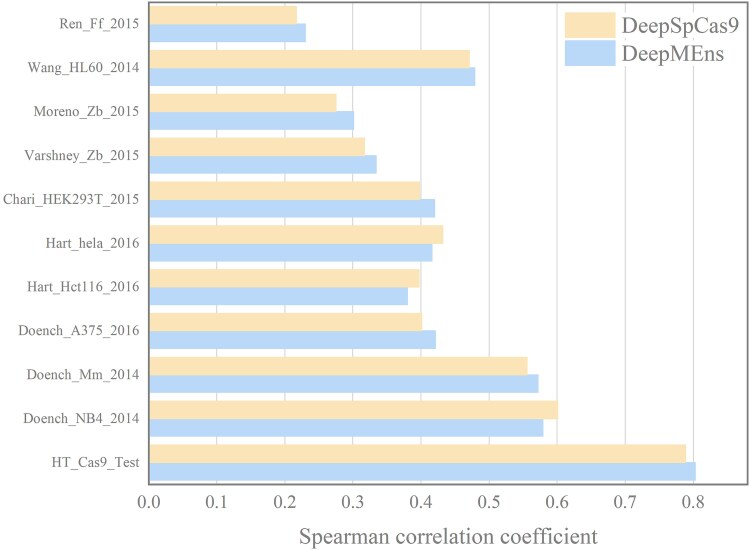
Comparison results between DeepMEns and DeepSpCas9 on the same training and independent test datasets.

Additionally, we trained DeepMEns model utilizing the dataset sourced from DeepSpCas9 [9], and subsequently validated its performance on ten independent test sets. As shown in Fig. 7, on the HT_Cas9_Test dataset, DeepMEns achieved a Spearman correlation coefficient of 0.803, which is 0.014 higher than that of DeepSpCas9. When evaluated on ten independent test datasets, DeepMEns averaged a Spearman score of 0.446, slightly exceeding DeepSpCas9’s score of 0.441, implying a degree of dominance. A detailed performance analysis of individual datasets demonstrated that DeepMEns outperformed DeepSpCas9 in seven datasets, while DeepSpCas9 was superior in only three. This comprehensive analysis indicates that our model exhibits significant robustness and generalization capabilities.

In summary, we conclude that DeepMEns showed slightly superior performance compared to other predictors based on three main factors. First, DeepMEns not only introduces sequence features but also DNA shape feature, secondary structure feature, positional encoding feature, and hand-crafted biological feature. Second, the combination of the attention mechanism with CNN and LSTM increases the predictive power and generalization ability of DeepMEns. Third, DeepMEns integrates several small models that can produce more accurate and reliable prediction, as indicated in previous studies [46].

### Ablation experiment

To validate the significance of each part in our proposed model, we conducted an ablation analysis by systematically removing one component at a time: (1) DeepMEns without hand-crafted biological features; (2) DeepMEns without one-hot and secondary structure features (the left part); (3) DeepMEns without DNA shape structure (the middle part); and (4) DeepMEns without position encoding (the right part). Through analysis of ablation experiment, we could assess the contribution of each type of feature representation. As shown in Table 3, the ablation analysis revealed that the exclusion of any part caused a decline in model performance. Notably, removing the left part led to the greatest drop in Spearman scores for the three independent test datasets, with reductions of 0.045, 0.063, and 0.065, respectively. This decline is likely due to the one-hot encoding and secondary structure capturing crucial internal sequence information, while the CNN with Transformer encoder enhances the model’s ability to process information from the raw sgRNA sequence.

**Table 3:** Ablation analysis of DeepMEns on WT-SpCas9, eSpCas9(1.1), and SpCas9-HF1 dataset test datasets.

Method	WT-SpCas9	eSpCas9(1.1)	SpCas9-HF1
DeepMEns	0.880	0.875	0.866
Without hand-crafted biological features	0.873	0.871	0.857
Without left part	0.835	0.812	0.801
Without middle part	0.876	0.872	0.863
Without right part	0.879	0.873	0.864

The removal of the middle part resulted in a minimal decrease in Spearman scores, by only 0.004, 0.003, and 0.003, indicating that DNA shape characteristics do influence editing efficiency. As anticipated, excluding the right part caused a slight drop in Spearman scores of 0.001, 0.002, and 0.002, likely due to the one-dimensional nature of position encoding, which compensates for the spatial information missing from the other two encodings. Additionally, the absence of hand-crafted biological features significantly impacted model performance, leading to decreases in Spearman scores of 0.007, 0.004, and 0.009, respectively. These findings underscore the critical role of each component in the DeepMEns model, demonstrating that the integration of diverse information types enhances sgRNA targeting effectiveness.

### DNA shape feature analysis

Because the contribution of shape features to the model is relatively small, we analyzed each type of shape feature to test whether the performance of the model can be further improved. First, we considered the predictive performance of the model when using shape features alone, and the performance was evaluated after excluding one type of shape feature and retaining the other four shape features. Table 4 shows the results of five situations for the three independent datasets of WT-SpCas9, eSpCas9(1.1), and SpCas9-HF1. When all DNA shape features were considered together, the model achieved Spearman scores of 0.834, 0.827, and 0.815 for the three datasets, respectively. Thus, it was observed that the exclusion of Roll dramatically reduced the performance of the model with Spearman scores of 0.773. 0.780, and 0.775, followed by Spearman scores of 0.802, 0.806, and 0.787 for without_HeIT; Spearman scores of 0.813, 0.807, and 0.801 for without_MGW; Spearman scores of 0.815, 0.807, and 0.805 for without_ProT, and Spearman scores of 0.809, 0.807, and 0.781, respectively, for without_EP. This result implied that the exclusion of each type of shape feature resulted in varying degrees of decrease in model performance; hence, we retained all shape features in our predictor.

**Table 4:** Analysis results of each kind of DNA shape feature.

Dataset	WT-SpCas9	eSpCas9(1.1)	SpCas9-HF1
Without_Roll	0.773	0.780	0.775
Without_ProT	0.815	0.807	0.805
Withour_MGW	0.813	0.807	0.801
Without_HeIT	0.802	0.806	0.787
Without_EP	0.809	0.807	0.781
All	0.834	0.827	0.815

## Conclusion

In the present study, a novel deep learning model, DeepMEns, was introduced to predict sgRNA on-target activity. DeepMEns is an ensemble of five sub-regression models, each comprising three parts, each part is based on different coding methods and network frameworks. Because of the multiple feature strategy, ensemble scheme, and attention mechanism, DeepMEns achieved an optimal predictive performance for three independent test datasets of WT-SpCas9, eSpCas9(1.1), and SpCas9-HF1 and for 6 of 10 independent test datasets from CRISeq as compared to other predictors. The ablation analysis also showed the ensemble strategy may improve the performance of the prediction model.

**Key Points**

- We introduce a novel ensemble deep learning algorithm for identifying sgRNA on-target activity through five base regression models.
- Each base regression model is composed of three network frameworks using different sequence encodings.
- We confirm that our predictor demonstrates robust performance on 13 independent test datasets.

## Supplementary Material

Supplementary_files_elae043

## Code availability

The DeepMEns core code are available from: https://github.com/Dingshumei/DeepMEns.git.

## Author Biographies

**Shumei Ding** is currently studying in the School of Science at Dalian Maritime University, China. Her main research interests are bioinformatics, computational biology, and deep learning.

**Jia Zheng** is a lecturer in the college of science, Dalian Maritime University, China. She obtained her PhD degree in the school of Mathematical Sciences from Zhejiang University in 2015. Her major research interests include mathematical modelling in bioinformatics and machine learning.

**Cangzhi Jia** received a PhD degree from the School of Mathematical Sciences, Dalian University of Technology, China. She is currently a professor in the School of Science, Dalian Maritime University, China. Her research interests include mathematical modeling in bioinformatics and machine learning.
