# Supplementary material for: DeepMEns: an ensemble model for predicting sgRNA on-target activity based on multiple features
Source: Brief Funct Genomics. 2024 Nov 11;24:elae043. doi: 10.1093/bfgp/elae043 (PMC11735754; doi:10.1093/bfgp/elae043)
Supplement: Supplementary_files_elae043 [file supplementary_files_elae043.docx]

Table S1. Detailed hand-crafted biological features.

| Hand-crafted biological feature | Dimension |
| --- | --- |
| structural feature (stem, dG, dG_binding_20, dG_binding_7to20 ) | 4 |
| GC content features (GC_above_10, GC_below_10, GC_count) | 3 |
| thermodynamics features (Tm global_False, 5mer_end_False, 8mer_middle_False, 4mer_start_False) | 4 |
| Zcurve | 3 |
| (A+T)/(C+G) ratio | 1 |
| GC/AT skew | 2 |

Table S2. Hyperparameters of DeepMEns and the corresponding search space.

| Hyperparameter | Search space | WT-SpCas9 | eSpCas9(1.1) | SpCas9-HF1 |
| --- | --- | --- | --- | --- |
| Filter number | {10, 20, 30, 40, 50} | 30 | 30 | 40 |
| Convolutional kernel size | {(1,2,3),(1,2,4),(1,2,5),(1,3,4),(1,3,5),(2,3,4),(2,3,5),(3,4,5)} | (1,3,5) | (1,4,5) | (1,3,5) |
| BiLSTM unit number | {16, 32, 64, 128} | 32 | 64 | 32 |
| Optimizer | {Adam, SGD,RMSprop, Adamax, Adagrad} | Adam | Adam | Adam |

Table S3. The Spearman Correlation Coefficient ($\rho$), Pearson Correlation Coefficient ($\gamma$) and Mean Square Error ($\mathrm{MSE}$) of DeepMEns model and its variants on WT-SpCas9, eSpCas9(1.1) and SpCas9-HF1 dataset test sets.

| Method | WT-SpCas9 | | | eSpCas9(1.1) | | | SpCas9-HF1 | | |
| --- | --- | --- | --- | --- | --- | --- | --- | --- | --- |
|  | $\rho$ | $\gamma$ | $MSE$ | $\rho$ | $\gamma$ | $MSE$ | $\rho$ | $\gamma$ | $MSE$ |
| Model1 | 0.860 | 0.891 | 0.0101 | 0.863 | 0.853 | 0.0094 | 0.851 | 0.869 | 0.0105 |
| Model2 | 0.869 | 0.900 | 0.0091 | 0.861 | 0.851 | 0.0096 | 0.855 | 0.873 | 0.0102 |
| Model3 | 0.870 | 0.903 | 0.0088 | 0.863 | 0.852 | 0.0095 | 0.850 | 0.866 | 0.0106 |
| Model4 | 0.871 | 0.901 | 0.0090 | 0.855 | 0.845 | 0.0098 | 0.853 | 0.869 | 0.0104 |
| Model5 | 0.870 | 0.903 | 0.0089 | 0.861 | 0.851 | 0.0098 | 0.852 | 0.869 | 0.0105 |
| DeepMEns | 0.880 | 0.911 | 0.0081 | 0.875 | 0.865 | 0.0087 | 0.866 | 0.882 | 0.0095 |

Table S4. The Spearman Correlation Coefficient($\rho$), Pearson Correlation Coefficient($\gamma$) and Mean Square Error($MSE$) of DeepMEns model and its sub-regression models on 10 independent datasets.

| Datasets | Methond | Model1 | Model2 | Model3 | Model4 | Model5 | DeepMEns |
| --- | --- | --- | --- | --- | --- | --- | --- |
| Doench_NB4_2014 | $\rho$ | 0.57 | 0.599 | 0.582 | 0.538 | 0.56 | 0.583 |
|  | $\gamma$ | 0.552 | 0.58 | 0.556 | 0.518 | 0.537 | 0.56 |
|  | $MSE$ | 0.1001 | 0.0983 | 0.0974 | 0.1054 | 0.0967 | 0.0986 |
| Doench_Mm_2014 | $\rho$ | 0.554 | 0.565 | 0.546 | 0.522 | 0.531 | 0.558 |
|  | $\gamma$ | 0.518 | 0.54 | 0.512 | 0.481 | 0.485 | 0.518 |
|  | $MSE$ | 0.1123 | 0.1099 | 0.1113 | 0.1204 | 0.1102 | 0.1119 |
| Doench_A375_2016 | $\rho$ | 0.389 | 0.405 | 0.38 | 0.384 | 0.387 | 0.400 |
|  | $\gamma$ | 0.349 | 0.378 | 0.343 | 0.351 | 0.347 | 0.362 |
|  | $MSE$ | 3.0631 | 3.0470 | 3.0656 | 3.0545 | 3.0842 | 3.0618 |
| Hart_Hct116_2016 | $\rho$ | 0.365 | 0.384 | 0.384 | 0.363 | 0.377 | 0.385 |
|  | $\gamma$ | 0.349 | 0.374 | 0.369 | 0.357 | 0.366 | 0.375 |
|  | $MSE$ | 3.629 | 3.5435 | 3.5063 | 3.5548 | 3.587 | 3.5613 |
| Hart_hela_2016 | $\rho$ | 0.3942 | 0.4003 | 0.399 | 0.398 | 0.412 | 0.413 |
|  | $\gamma$ | 0.383 | 0.392 | 0.393 | 0.394 | 0.401 | 0.405 |
|  | $MSE$ | 2.8759 | 2.8226 | 2.7983 | 2.8165 | 2.8462 | 2.829 |
| Chari_HEK293T_2015 | $\rho$ | 0.395 | 0.404 | 0.418 | 0.408 | 0.408 | 0.413 |
|  | $\gamma$ | 0.356 | 0.368 | 0.379 | 0.371 | 0.37 | 0.376 |
|  | $MSE$ | 0.974 | 0.9327 | 0.9207 | 0.9358 | 0.9479 | 0.9398 |
| Varshney_Zb_2015 | $\rho$ | 0.337 | 0.322 | 0.317 | 0.34 | 0.361 | 0.334 |
|  | $\gamma$ | 0.355 | 0.365 | 0.345 | 0.335 | 0.362 | 0.355 |
|  | $MSE$ | 0.1565 | 0.1671 | 0.1698 | 0.1738 | 0.1611 | 0.1643 |
| Moreno_Zb_2015 | $\rho$ | 0.271 | 0.266 | 0.278 | 0.277 | 0.277 | 0.276 |
|  | $\gamma$ | 0.259 | 0.257 | 0.263 | 0.264 | 0.266 | 0.265 |
|  | $MSE$ | 0.1273 | 0.1336 | 0.1326 | 0.1351 | 0.1265 | 0.130 |
| Wang_HL60_2014 | $\rho$ | 0.485 | 0.486 | 0.464 | 0.453 | 0.474 | 0.483 |
|  | $\gamma$ | 0.451 | 0.458 | 0.432 | 0.421 | 0.436 | 0.448 |
|  | $MSE$ | 0.022 | 0.0249 | 0.0253 | 0.0238 | 0.0235 | 0.0229 |
| Ren_Ff_2015 | $\rho$ | 0.149 | 0.136 | 0.136 | 0.23 | 0.148 | 0.181 |
|  | $\gamma$ | 0.17 | 0.149 | 0.169 | 0.18 | 0.124 | 0.163 |
|  | $MSE$ | 0.125 | 0.109 | 0.0998 | 0.1063 | 0.1135 | 0.1069 |
| All | Mean $\rho$ | 0.391 | 0.397 | 0.390 | 0.391 | 0.394 | 0.403 |
|  | Mean $\gamma$ | 0.374 | 0.386 | 0.376 | 0.367 | 0.370 | 0.383 |
|  | STEDV $\rho$ | 0.127 | 0.137 | 0.129 | 0.097 | 0.120 | 0.122 |
|  | STDEV $\gamma$ | 0.113 | 0.125 | 0.111 | 0.098 | 0.115 | 0.114 |
